# No longer diseases of the wealthy: prevalence and health-seeking for self-reported chronic conditions among urban poor in Southern India

**DOI:** 10.1186/1472-6963-13-306

**Published:** 2013-08-13

**Authors:** Upendra Bhojani, Thriveni S Beerenahalli, Roopa Devadasan, CM Munegowda, Narayanan Devadasan, Bart Criel, Patrick Kolsteren

**Affiliations:** 1 Institute of Public Health, 250, 2 C Cross, 2 C Main, Girinagar, First Phase, Bangalore 560085, Karnataka, India; 2 Department of Public Health, Institute of Tropical Medicine, Nationalestraat 155, 2000 Antwerp, Belgium; 3 Department of Public Health, Ghent University, De Pintelaan 185, Block A, B- 9000 Ghent, Belgium

**Keywords:** Chronic conditions, Slum, Healthcare seeking, Prevalence, Non-communicable diseases, Urban poor, India

## Abstract

**Background:**

The burden of chronic conditions is high in low- and middle-income countries and poses a significant challenge to already weak healthcare delivery systems in these countries. Studies investigating chronic conditions among the urban poor remain few and focused on specific chronic conditions rather than providing overall profile of chronic conditions in a given community, which is critical for planning and managing services within local health systems. We aimed to assess the prevalence and health- seeking behaviour for self-reported chronic conditions in a poor neighbourhood of a metropolitan city in India.

**Methods:**

We conducted a house-to-house survey covering 9299 households (44514 individuals) using a structured questionnaire. We relied on self-report by respondents to assess presence of any chronic conditions, including diabetes and hypertension. Multivariable logistic regression was used to analyse the prevalence and health-seeking behaviour for self-reported chronic conditions in general as well as for diabetes and hypertension in particular. The predictor variables included age, sex, income, religion, household poverty status, presence of comorbid chronic conditions, and tiers in the local health care system.

**Results:**

Overall, the prevalence of self-reported chronic conditions was 13.8% (95% CI = 13.4, 14.2) among adults, with hypertension (10%) and diabetes (6.4%) being the most commonly reported conditions. Older people and women were more likely to report chronic conditions. We found reversal of socioeconomic gradient with people living below the poverty line at significantly greater odds of reporting chronic conditions than people living above the poverty line (OR = 3, 95% CI = 1.5, 5.8). Private healthcare providers managed over 80% of patients. A majority of patients were managed at the clinic/health centre level (42.9%), followed by the referral hospital (38.9%) and the super-specialty hospital (18.2%) level. An increase in income was positively associated with the use of private facilities. However, elderly people, people below the poverty line, and those seeking care from hospitals were more likely to use government services.

**Conclusions:**

Our findings provide further evidence of the urgent need to improve care for chronic conditions for urban poor, with a preferential focus on improving service delivery in government health facilities.

## Background

The rising burden of chronic conditions has drawn the attention of public health researchers and policy makers worldwide. Estimates indicate that chronic conditions will cause 41 million deaths in 2015 [1]. The chronic condition burden is very high in low- and middle-income countries, where over 80% of deaths from chronic conditions have been estimated to occur [1]. In India, chronic conditions are the leading cause of death. These conditions have been estimated to have caused 53% of all deaths in India in 2005 and are projected to account for 66.7% of all deaths by 2020 [2,3].

In an era of worsening health inequities, it is important to highlight the issues faced by vulnerable communities. Recent studies report a high burden from chronic conditions and chronic condition risk factors among the urban poor in low- and middle-income countries, including India [4-9]. The unfavourable social determinants in health and inequities in access to healthcare leave the urban poor in India with dismal health indicators [10]. With rapid urbanisation, the number of urban poor, including slum dwellers, is also on rise. According to the most recent estimates available for the urban Indian population, 26.3% of urban Indians live in slums and 25.7% live below the poverty line [11,12]. However, studies investigating chronic conditions among the urban poor remain few in India, particularly for the southern part of the country.

Furthermore, most of the studies in India report the prevalence of specific chronic conditions. Very few studies provide an overall prevalence and profile of chronic conditions in a given community [13,14]. Such information is critical for planning and managing services within local health systems, particularly when desirable health system characteristics for the effective prevention and management of any chronic condition are known (e.g., continuity of care, financial protection, active involvement of patients) [15,16].

In this study, we aimed to assess the prevalence and health-seeking behaviour for self-reported chronic conditions in general as well as for diabetes and hypertension in particular, in a poor neighbourhood of a metropolitan city in South India. We also examined the association of these outcomes with several predictor variables.

## Methods

### Study setting

This study was conducted in Kadugondanahalli (KG Halli), one of the 198 administrative units of Bangalore city, the metropolitan capital of the state of Karnataka. Municipal government records indicate that KG Halli has a population of nearly 35000 people in an area of 0.7 square kilometres. KG Halli has one recognised slum area. The population in KG Halli is comprised of natives as well as migrants from other Indian states. The population is comprised of people who speak five different languages and represent all major religions in India.

KG Halli has a mixed healthcare delivery system with two government health centres and at least 32 private health facilities. Private health facilities are composed of single-doctor clinics and hospitals. Private providers work on fee-for-service basis and have been trained in different systems of medicines: Unani, Ayurveda and modern allopathic medicine [17]. This pluralistic nature of the health care delivery system is a characteristic feature of the Indian health system. Irrespective of the training received, the majority of KG Halli private providers either practice modern medicine or a mix of systems. The provincial and municipal governments run two health centres in KG Halli that mainly provide outpatient care and outreach services. The services provided by these two health centres are free for people living below the poverty line, with nominal user-fees for selected services for other patients.

### Data collection and measurements

We conducted a house-to-house survey in KG Halli between June 2009 and March 2010 to establish a baseline for the Urban Health Action Research Project (UHARP). This project is being implemented by the Institute of Public Health Bangalore. The UHARP aims to work with residents of KG Halli, local health services (government and private) and health authorities to improve the quality of healthcare for the residents of KG Halli.

A structured questionnaire, initially developed in English and later translated into the local language (*Kannada*), was used to collect data on socio-demography, self-reported illness profile, health-seeking behaviour, and healthcare expenditures. The questionnaire was field-tested on 50 households and subsequently refined. Five trained data collectors who were fluent in languages commonly spoken in the area administered the questionnaire at the household level. As most adults in the area would go out for work for most of the day, any family member aged 18 years or above was considered an eligible respondent.

For the analysis of the prevalence of chronic conditions, three binary outcome variables were defined. These were the ‘absence’ (coded as ‘0’) or ‘presence’ (coded as ‘1’) of the following: i) any chronic condition, ii) diabetes, and iii) hypertension. A chronic condition is defined as an illness or impairment that lasts for a long duration. The minimum time period for an illness to be considered chronic varies depending on the source of the definition, ranging from three months to one year [18,19]. We considered a chronic condition to be present when a respondent reported taking medications on a daily basis for at least the 30 days preceding the survey. Respondents often reported cases where their family members were prescribed regular medication by a healthcare provider but were unable to take the medication for various reasons. We recorded such instances as the presence of chronic conditions. The names of chronic conditions were initially recorded using the lay terms reported by respondents and later revised by researchers to categorise them, to the extent possible, into specific conditions (e.g., diabetes was often referred to as ‘sugar’). Based on the names of the reported chronic conditions, the presence or absence of diabetes and hypertension were also recorded.

Predictor variables were chosen based on earlier evidence, theoretical knowledge, and the availability of the variables in the KG Halli house-to-house survey. Earlier studies have associated the prevalence of self-reported chronic conditions with age, sex, income, education, and religion [13,20,21]. As predictor variables, we included sex (‘male’ or ‘female’), age (in years and transformed into three age groups: ‘≤19’, ‘20-39’, ‘≥40’ year), per capita income per month (as income quintiles), religion (‘Islam’, ‘Hinduism’, and ‘Christianity’), and the household poverty status (‘above’ or ‘below’ the poverty line), as established by the type of ration card possessed by the household. A ration card is a document issued to households by government authorities to enable access to essential commodities at subsidised rates and has also become an important identity card for the official poverty status of households in India.

For the analysis of the health-seeking behaviour, three binary outcome variables were defined. These were type of health services sought (‘private’ coded as ‘0’, ‘government’ coded as ‘1’) for the following: i) a chronic condition, ii) diabetes, and iii) hypertension. However, in India, patients often use government and private health facilities simultaneously, even for a single episode of a chronic condition. For this study, we coded the outcome variable based on the nature of the health facility through which the patient “entered” the health system. In other words, we coded the variable based on the nature of the health facility where the first consultation occurred. For example, when a person with a chronic condition approaches a government health centre for a first consultation, he/she might be asked to buy medicines from a private pharmacy if the prescribed medicines are not available at that centre. In such a case, the health-seeking behaviour would be coded as ‘1’ (‘government health service’).

All the predictor variables described earlier, in case of prevalence estimation, were included with a revised coding by individual age (‘<40’, ‘40-49’, ‘50-59’, and ‘>60’ years) that took into consideration the skewed age distribution among individuals who reported chronic conditions. In addition, two more predictor variables were included: i.e., the ‘presence’ or ‘absence’ of more than one chronic condition (comorbidity), and the tier of the healthcare services sought. Three tiers of healthcare services were defined based on where the person with a chronic condition was being managed at the time of the survey: i) ‘clinics/health centres’, ii) ‘referral hospitals’ with in-patient facilities, and iii) ‘super-specialty hospitals’ attached to medical schools. Though there are overlaps in the provision of services across clinics/health centres, referral hospitals, and super-specialty hospitals, they roughly correspond to primary, secondary and tertiary healthcare services, respectively.

### Ethics statement

At the time of this study, the Institute of Public Health, Bangalore did not have an Institutional Ethics Committee, and a policy requiring a formal ethics approval for non-clinical survey research. However, we followed ethical principles set for such research.

Due to the low literacy level and perceived reservations about signing documents among the KG Halli residents, an informed verbal consent was sought before data collection. Respondents received an explanation about the purpose of the survey, the voluntary nature of their participation, the privacy of data, and the anonymity of respondents and family members in a language that they were comfortable with.

### Data analysis

The data were entered using EpiData Entry software (The EpiData Association, Odense, Denmark). Data were externally validated through revisiting the households and confirming the responses for 20% of randomly selected completed questionnaires. The data were checked for errors and missing values before being analysed using STATA 11.2 (StataCorp, Texas, USA).

The prevalence of self-reported chronic conditions is reported as a percentage with 95% confidence interval. To identify the predictors of self-reported chronic conditions, a multivariable logistic regression model was developed using all aforementioned predictors. The interaction between predictor variables was checked and two-way interaction terms that were significant at *p* < 0.05 were included in a multivariable model. Similar to a backward elimination technique, the predictors that were not significant at *p* < 0.05 were then dropped individually, and the resultant models were compared for goodness of fit (using a likelihood-ratio test) until no further improvement was possible. A similar process was used to develop the final multivariable models for all other outcome variables. We checked for and excluded the presence of multi-colinearity using post-estimation commands. The final models are presented with the adjusted odds ratio (OR), 95% confidence interval, and *p* values.

## Results

We received responses from 98.5% (9299) of households (44514 individuals). Non-response was either due to refusal to respond (0.3%) or the absence of household members (1.2%) on the follow-up visit by data collectors. The socio-demographic characteristics of the sample population are presented in Table 1.

**Table 1:** Socio-demographic characteristics of the sample population

**Sex** n(%)	
Male	22702 (51.0)
Female	21801 (49.0)
**Age groups** n(%)	
≤19 years	17335 (39.0)
20-39 years	17140 (38.5)
≥40 years	10013 (22.5)
**Per capita income per month in INR** Median (inter-quartile range)	
First quintile	1200 (1000, 1285.7)
Second quintile	1625 (1500, 1750)
Third quintile	2000 (2000, 2250)
Fourth quintile	2875 (2531.3, 3200)
Fifth quintile	5000 (4000, 6142.9)
**Religion** n(%)	
Islam	30481 (68.7)
Hinduism	9317 (21.0)
Christianity	4569 (10.3)
Household poverty status* n(%)	
Above the poverty line	23442 (52.7)
Below the poverty line	4783 (10.7)

n = 44514 individuals. *Total does not add up to 100 because 36.6% individuals (their households) did not possess a ration card.

**Figure 1:**
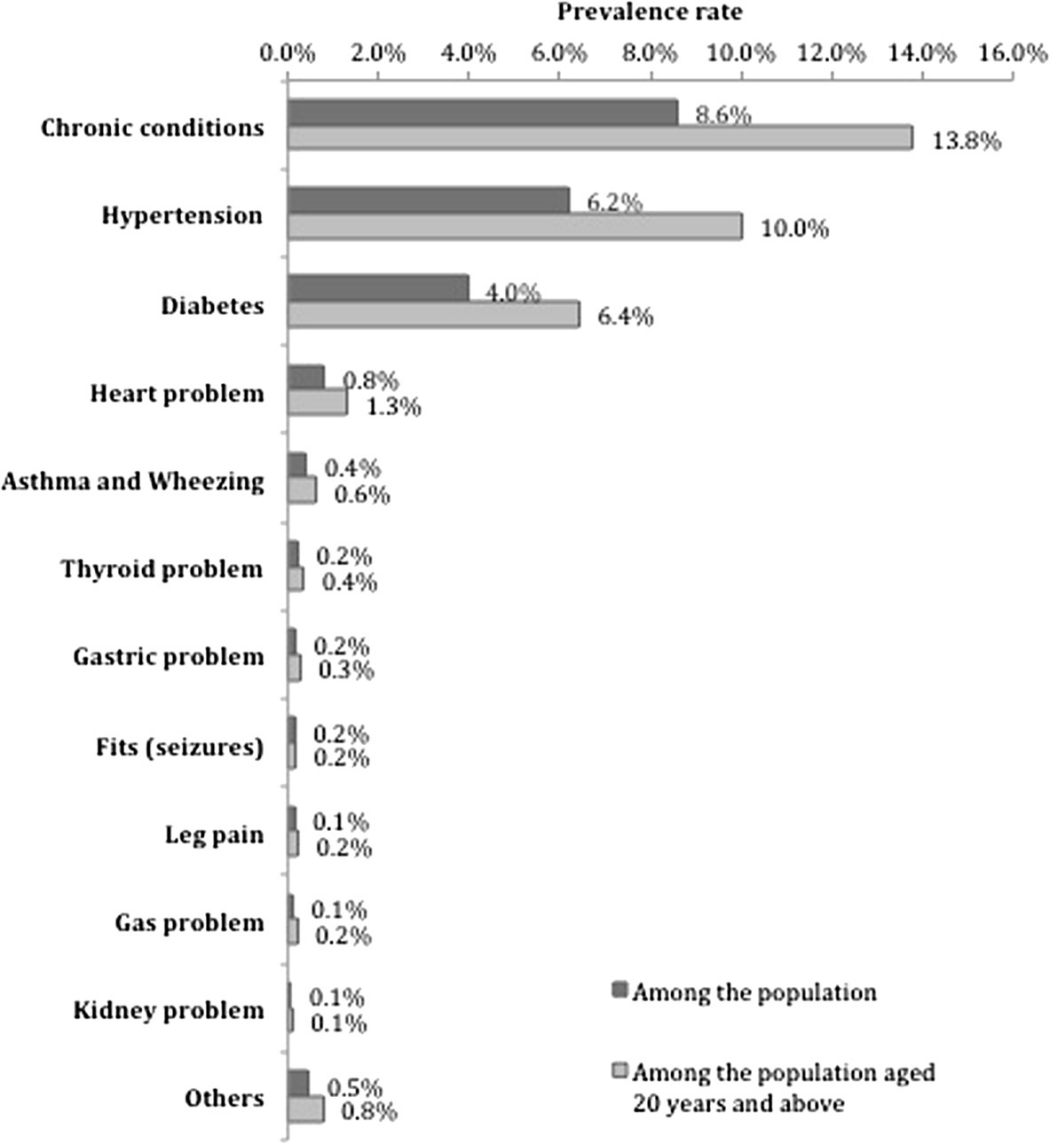
**Prevalence rate for self-reported chronic conditions (n = 44514).** This figure provides prevalence rate for any self-reported chronic condition in general as well as for several specific self-reported chronic condition in particular.

The prevalence of various self-reported chronic conditions in KG Halli is presented in Figure 1. The prevalence of self-reported chronic conditions was 8.6% (95% CI = 8.4, 8.9) in the general population and 13.8% (95% CI = 13.4, 14.2) among adults (age ≥20 years). The two most commonly reported conditions were hypertension and diabetes, with a self-reported prevalence of 10.0% and 6.4%, respectively, among adults. Overall, 4.5% (95% CI = 4.3, 4.8) of people reported having at least two chronic conditions. The presence of an additional chronic condition was reported by 57.4% of people with diabetes and 43% of people with hypertension.

The results of the multivariable logistic regression for chronic conditions are presented in Table 2. Women were 3.2 times more likely to report a chronic condition than men (*p* < 0.001). People in older age groups were more likely to report chronic conditions than people 19 years old or younger (*p* < 0.001). Increases in per capita income had an inverse graded relationship with the overall prevalence of self-reported chronic conditions. A similar trend was observed for diabetes, but the association was not statistically significant. In the case of self-reported hypertension, the reduction in prevalence was significant only for the two uppermost income quintiles (*p* < 0.05). While people living in households below the poverty line were more likely to report the presence of a chronic condition (including hypertension) compared with households above the poverty line (*p* < 0.005), it was the opposite pattern for diabetes reports (*p* < 0.001).

**Table 2:** Predictors of self-reported chronic conditions

**Predictor variables**	**Overall chronic conditions**		**Diabetes**		**Hypertension**	
	Adjusted odds ratio* (95% CI)	***p *value**	Adjusted odds ratio* (95% CI)	***p *value**	Adjusted odds ratio* (95% CI)	***p *value**
**Sex**						
Male	-	-	-	-	-	-
Female	3.2 (2.6, 4.0)	<0.001	2.5 (1.8, 3.5)	<0.001	4.6 (3.6, 5.8)	<0.001
**Age groups (years)**						
≤19	-	-	-	-	-	-
20-39	6.7 (4.8, 9.5)	<0.001	10.9 (4.9, 24.0)	<0.001	12.2 (7.3, 20.3)	<0.001
≥40	58.8 (36.3, 95.2)	<0.001	106.8 (40.7, 280.2)	<0.001	116.1 (59.5, 226.4)	<0.001
**Monthly per capita income**						
First quintile	-	-	-	-	-	-
Second quintile	0.8 (0.6, 1.0)	0.047	0.8 (0.5, 1.2)	0.226	0.8 (0.6, 1.1)	0.211
Third quintile	0.5 (0.3, 0.8)	0.002	0.5 (0.2, 1.1)	0.097	0.6 (0.4, 1.0)	0.056
Fourth quintile	0.4 (0.2, 0.7)	0.001	0.3 (0.1, 1.1)	0.072	0.4 (0.2, 0.9)	0.023
Fifth quintile	0.2 (0.1, 0.5)	<0.001	0.2 (0.1, 1.1)	0.072	0.3 (0.1, 0.9)	0.026
**Household poverty status**						
Above the poverty line	-	-	-	-	-	-
Below the poverty line	3.0 (1.5, 5.8)	0.002	0.6 (0.5, 0.7)	<0.001	1.9 (0.7, 4.9)	0.196
**Religion**						
Islam	-	-	-	-	-	-
Hinduism	0.9 (0.8, 1.1)	0.227	1.0 (0.8, 1.1)	0.527	0.9 (0.8, 1.1)	0.177
Christianity	1.2 (1.0, 1.5)	0.078	1.0 (0.8, 1.2)	0.665	1.2 (0.9, 1.5)	0.175
**Interaction terms**						
Sex*Religion	0.7 (0.6, 0.8)	<0.001			0.7 (0.6, 0.8)	<0.001
Sex* Monthly per capita income			0.8 (0.8, 0.9)	<0.001		
Age group*Monthly per capita income	1.1 (1.1, 1.2)	<0.001	1.2 (1.0, 1.4)	0.007	1.1 (1.0, 1.2)	0.019
Age group*Household poverty status	0.6 (0.5, 0.8)	<0.001			0.7 (0.5,1.0)	0.039

- Referent category. *Adjusted odds ratio as obtained from multivariable logistic regression models. All the predictor variables were included in the initial model, including two-way interaction terms that were significant at *p* < 0.05 during binominal logistic regression. Similar to a backward elimination technique, the predictors that were not significant at *p* < 0.05 were then dropped individually, and the resultant models were compared for goodness of fit (using a likelihood-ratio test) until no further improvement was possible.

Some two-way interactions between predictor variables were significant (Table 2). A gender-stratified multivariable analysis (detailed data not presented in this paper) revealed that religion was a significant predictor of chronic conditions overall and of hypertension among women. Muslim women were more likely to report chronic conditions compared with Hindu (OR = 0.6, *p* < 0.001) and Christian (OR = 0.7, *p* < 0.001) women. Although per capita income was not a significant predictor for self-reported diabetes prevalence among the population, per capita income did turn out to be a significant predictor for men, with poor men being at higher risk of reporting diabetes (*p* < 0.05). Similarly, a multivariable analysis stratified by age groups revealed that the per capita income was a significant positive predictor for self-reported diabetes prevalence but only for patients 40 years old and older (OR = 1.4, *p* = 0.001).

The socio-demographic information and self-reported health-seeking behaviour for people with chronic conditions is summarised in Table 3. Overall, 80.6% (95% CI = 79.3, 81.8) of people with chronic conditions sought care from private healthcare providers, while 19.4% (95% CI = 18.1, 20.7) sought care from government health services. A similar trend was found for diabetes and hypertension. The majority of people with a chronic condition received care from clinics/health centres (42.9%, 95% CI = 41.5, 44.5), followed by referral hospitals (38.9%, 95% CI = 37.3, 40.4) and super-specialty hospitals (18.2%, 95% CI = 17.0, 19.5). A similar trend was observed with hypertension, while in the case of diabetes, care was most commonly sought from referral hospitals, followed by clinics/health centres and super-specialty hospitals.

**Table 3:** Characteristics of population with self-reported chronic conditions

	**People with chronic conditions (n = 3844)**	**People with diabetes (n = 1760)**	**People with hypertension (n = 2756)**
**Sex** n(%)			
Male	1533 (39.9)	785 (44.5)	942 (34.1)
Female	2308 (60.1)	973 (55.6)	1810 (65.9)
**Age** (years) Mean (SD)	50.2 (14.1)	52.9 (12)	51.1 (13.7)
**Age groups** n(%)			
≤19 years	83 (2.2)	9 (0.5)	36 (1.3)
20-39 years	99 (2.6)	12 (0.7)	45 (1.6)
≥40 years	3123 (81.3)	1567 (89.1)	2278 (82.8)
**Income per capita per month** (INR) Median (inter-quartile range)			
First quintile	1200 (1000, 1333.3)	1170.8 (966.7, 1285.7)	1200 (1000, 1333.3)
Second quintile	1650 (1500, 1750)	1666.7 (1500, 1727.3)	1666.7 (1500, 1750)
Third quintile	2000 (2000, 2250)	2000 (2000, 2250)	2090.9 (2000, 2250)
Fourth quintile	2857.1 (2500, 3166.7)	2857.1 (2538.5, 3200)	2857.1 (2500, 3154.8)
Fifth quintile	5000 (4000, 6428.6)	5000 (4000, 6250)	5000 (4000, 6464.3)
**Religion** n(%)			
Islam	2612 (68.0)	1144 (65.1)	1893 (68.9)
Hinduism	798 (20.8)	401 (22.8)	566 (20.5)
Christianity	430 (11.2)	213 (12.0)	292 (10.6)
Household poverty status* n(%)			
Above the poverty line	2404 (62.5)	1156 (65.7)	1730 (62.8)
Below the poverty line	275 (7.2)	106 (6.0)	191 (6.9)
**Presence of comorbidity** n(%)	1218 (31.7)	1011 (57.4)	1184 (42.9)
**Type of health service sought** n(%)			
Government	724 (19.4)	258 (14.8)	485 (18.1)
Private	3005 (80.6)	1483 (85.2)	2172 (81.9)
**Tiers of health services sought** n(%)			
Clinics/ health centres	1600 (42.9)	624 (36.0)	1287 (48.5)
Referral hospitals	1449 (38.9)	853 (49.0)	971 (36.6)
Super-specialty hospitals	680 (18.2)	264 (15.0)	399 (14.9)

*Total does not add up to 100 because some of the individuals (their households) did not possess a ration card.

People in older age groups were more likely to report to seek care from government health services (Table 4). For diabetes, the likelihood of seeking care from government health services increased consistently throughout the age groups (*p* < 0.05). In the case of hypertension, this increase was statistically significant only for people aged 60 years or above. With an increase in per capita income, people were more likely to report seeking care from private providers, except for people seeking diabetes care. People were more likely to report seeking care from government services when they utilised referral hospitals and super-specialty hospitals, compared with those utilising clinics/health centres. In general, people living below the poverty line were more likely to report the utilisation of government health services. Such an association between poverty and utilisation of government services was not statistically significant for diabetes. A multivariable analysis stratified by age groups (detailed data not presented in this paper) revealed an interaction between age groups and the health facilities tiers sought by patients. In general, the positive association between age group and self-reported utilisation of government facilities for chronic-condition care was significant only for patients over the age of 60 years (Table 4). The association was statistically significant for all age groups seeking care at super-specialty hospitals. The size effect of the positive association decreased with increases in age.

**Table 4:** Predictors of seeking care from government health services (opposed to private health services)

**Predictor variables**	**Overall chronic conditions (n = 3844)**		**Diabetes (n = 1760)**		**Hypertension (n = 2756)**	
	Adjusted odds ratio* (95% CI)	***p *value**	Adjusted odds ratio* (95% CI)	***p *value**	Adjusted odds ratio* (95% CI)	***p *value**
**Age groups (years)**						
≤40	-	-	-	-	-	-
40-50	1.1 (0.7, 1.8)	0.584	5.3 (1.6, 17.3)	0.006	1.2 (0.7, 2.0)	0.599
50-60	1.7 (0.9, 3.1)	0.106	13.5 (2.7, 67.5)	0.002	1.6 (0.8, 3.4)	0.175
≥60	3.7 (1.6, 8.3)	0.002	40.2 (5.0,325.7)	0.001	3.4 (1.3, 8.8)	0.010
**Monthly per capita income**						
First quintile	-	-	-	-	-	-
Second quintile	0.7 (0.5, 1.0)	0.028	0.7 (0.4, 1.2)	0.235	0.4 (0.2, 0.6)	<0.001
Third quintile	0.5 (0.3, 0.7)	0.001	0.6 (0.3, 1.1)	0.106	0.2 (0.1, 0.4)	<0.001
Fourth quintile	0.4 (0.2, 0.7)	0.001	1.1 (0.7, 1.9)	0.617	0.1 (0.1, 0.4)	<0.001
Fifth quintile	0.3 (0.1, 0.5)	<0.001	0.6 (0.3, 1.0)	0.066	0.1 (0.0, 0.3)	<0.001
**Household poverty status**						
Above the poverty line	-	-	-	-	-	-
Below the poverty line	1.4 (1.0, 2.0)	0.069	1.3 (0.7, 2.4)	0.392	5.2 (1.6, 17.1)	0.007
**Religion**						
Islam	-	-			-	-
Hinduism	0.8 (0.5, 0.1)	0.185			0.9 (0.5, 1.5)	0.676
Christianity	0.4 (0.2, 0.8)	0.011			0.3 (0.1, 0.8)	0.019
**Tiers of health services**						
Clinics/health centres	-	-	-	-	-	-
Referral hospitals	2.4 (1.5, 3.8)	<0.001	5.3 (1.9, 14.7)	0.001	1.6 (0.9, 3.0)	0.115
Super-specialty hospitals	30.3 (14.4, 63.8)	<0.001	99.9 (16.2, 614.1)	<0.001	9.2 (3.0, 28.2)	<0.001
**Interaction terms**						
Age group*Tiers of health services	0.8 (0.7, 0.9)	<0.001	0.6 (0.5, 0.9)	0.002	0.8 (0.7, 0.9)	0.008
Monthly per capita income *Religion	1.2 (1.0, 1.3)	0.010			1.2 (1.0, 1.4)	0.019
Monthly per capita income *Tiers of health service					1.1 (1.0, 1.3)	0.017
Household poverty status*Religion					0.4 (0.2, 0.9)	0.021

- Referent category. *Adjusted odds ratio as obtained from multivariable logistic regression models. All the predictor variables were included in the initial model, including two-way interaction terms that were significant at *p* < 0.05 during binominal logistic regression. Similar to a backward elimination technique, the predictors that were not significant at *p* < 0.05 were then dropped individually, and the resultant models were compared for goodness of fit (using likelihood-ratio test) until no further improvement was possible.

## Discussion

In this study, we found high prevalence of self-reported chronic conditions in a poor urban neighbourhood of the city of Bangalore, with hypertension and diabetes being the two most commonly reported conditions.

Our estimates of prevalence of self-reported diabetes and hypertension in KG Halli are comparable or higher than bio-medically derived estimates from slums in Bangladesh and Kenya [4,6]. To date, there have been very few epidemiological studies estimating the overall prevalence of chronic conditions specifically in slums or low-income regions. Even our conservative estimate (that largely excludes patients who were not on regular medication) of the overall prevalence of self-reported chronic conditions (8.6%) is nearly two times higher than the estimate reported by a study conducted in a slum in the western part of India seven years ago [14]. We found a much higher prevalence of self-reported hypertension and diabetes compared to the results of two earlier studies conducted in north and west Indian slums in Faridabad (hypertension 6.7%, diabetes 1.3%) and in Ahmedabad (hypertension 1%), respectively [7,14]. Understandably, the prevalence of self-reported hypertension and diabetes in our study was lower than the estimates using bio-medical diagnostics tools for hypertension (range: 11.6%, 16.5%) and for diabetes (range: 10.3%, 13.1%) from slums in different parts of the country [7,22,23]. Studies in India have indeed demonstrated that many people with hypertension and diabetes remain undiagnosed. The prevalence of undiagnosed diabetes in India is higher than diagnosed diabetes; thus, more people remain undiagnosed than those who self-report diabetes [24,25].

In KG Halli, older people and women were more likely to report chronic conditions. It is worrying to note that even among people in a relatively young and productive age group (20–39 years), the risk of any chronic condition, including diabetes and hypertension, was significantly higher than those younger than 19 years old (over six times higher for overall chronic conditions, over ten times higher for diabetes and/or hypertension).

A higher income had a negative association with the prevalence of self-reported chronic conditions. Generally, in the initial phase of epidemiologic transition, the affluent part of the population is affected more with chronic conditions, but once the transition progresses, the socio-economic gradient reverses, making the poor more vulnerable to chronic conditions. Among Southeast Asian countries, Thailand has already reported an inverse relation between income and the prevalence of self- reported chronic conditions [26]. There is an indication of a reversal of socioeconomic gradient for certain chronic conditions in India as well. Deepa et al. [27] demonstrated that in Chennai, over a period of ten years, the prevalence of self-reported diabetes among low-income groups increased more rapidly than among middle-income groups and became similar to that observed in middle-income groups. Other studies, conducted in the past five years, also report the prevalence of some self-reported chronic conditions (especially hypertension, diabetes, and asthma) in urban slums as similar or higher than that of the general urban population [7,8,22,23,28]. Our study builds on this early evidence and found a significant inverse relationship between income and the prevalence of overall self-reported chronic conditions (including hypertension) among the urban poor.

Our study found that Muslim women had greater odds of reporting chronic conditions. Rao et al. [21] reported that that Muslims in Karnataka had over two-fold higher odds of reporting diabetes compared with Hindus. In Andhra Pradesh (neighbouring Karnataka), a study demonstrated that Muslim women were at higher risk of being obese compared with women of other religions [29]. Religion-based differences in dietary patterns, including the higher consumption of meat-based products by Muslims, and social mobility restrictions on women might explain the observed findings [29-31].

In KG Halli, private healthcare providers managed over 80% of self-reported chronic conditions during the study period. These results are similar to the role played by the private sector in healthcare delivery at the national level. Overall, 81% of outpatient and 61.7% of hospitalisation episodes are managed in private-sector health care facilities [32]. The results of our study indicate that an increase in per capita income was associated with a greater likelihood of seeking care from private healthcare providers. Studies in India have shown a preference for private healthcare providers in general, and for chronic conditions in particular, among the urban poor and slum dwellers [14,33-36]. Various factors, including the proximity of health facility, short waiting time, lower fees (i.e., the ones charged by ‘informal’ providers), favourable opening/closing timings, patient satisfaction, and perceived effectiveness of treatment leading to a short recovery period, have been reported as reasons by people for seeking private providers [33,35-37].

Despite the general preference for private-sector health care, those in the extreme poverty depend on government health services. Our study indicates that people living below the poverty line were over five times more likely to report seeking care for hypertension from government health services compared with private services. Preference for government health services was also greater when referral hospitals and super-specialty hospitals were used. Those results can be explained by difficulties in affording private providers for such care. Furthermore, the elderly were more likely to report use of government facilities. This finding might be explained by the inequity in intra-household allocation of resources for healthcare and the neglect of the elderly [38-41]. The elderly are also more likely to have complications from chronic conditions and hence are more likely to need care at referral/super-specialty hospitals, which are expensive for patients seeking private care. These results indicate that government health services need to be strengthened, particularly in terms of providing care for chronic conditions, especially for the patients in poverty and the elderly.

### Study limitations

One of the limitations of our study is the use of respondents’ self-report as well as our operational definition of chronic conditions, which would exclude individuals who either remain undiagnosed or are not on daily medication, leading to an underestimate of the true prevalence of chronic conditions. In fact the degree of underestimation could be higher in our sample population, a low income setting, as it is known that KG Halli residents face financial constraints in accessing healthcare [42]. Nevertheless community-based prevalence estimates of self-reported chronic conditions, including diabetes and hypertension, are a crucial starting point in understanding the burden of these conditions. Such estimates are hardly available for poor neighborhoods in India. In fact, in resource-constrained settings, self-reported morbidity has been shown to be an important and valid measure of health [43].

For this study, we used a simple measure of health-seeking behaviour, i.e., the type of healthcare facility that was the initial location of healthcare consultation. However, it is important to remember that this is merely the entry point in the healthcare system. In reality, people’s health-seeking behaviour is complex and involves the mixed use of different provider systems during the treatment of a single episode of illness. For example, a person who uses a government health centre for medical consultation might (have to) use a private pharmacy or a private laboratory for respective services when seeking care for his/her episode of chronic condition. Finally, although our study findings from KG Halli might not be strictly and statistically generalised to all the other urban poor areas in the country, they indeed point towards a possible high burden of chronic conditions among urban poor in general and provide analytical guidance while studying such groups in India and in the region. In context of KG Halli, our findings would inform and shape the future strategies of the UHARP to improve the healthcare for KG Halli residents.

In general, our findings point to the need to improve the management of chronic conditions, including prevention, as part of the offerings of health services in urban poor areas. Unfortunately, the National Urban Health Mission proposed to be implemented between 2008–2012 by the federal government to revamp urban health systems, and especially to improve access of urban poor to health care services, remains yet to be implemented [44].

## Conclusions

We report a high prevalence of self-reported chronic conditions in the poor urban neighbourhood of KG Halli in the city of Bangalore. Our study builds on earlier evidence of a reversal of socio-economic gradient for chronic conditions by revealing a graded inverse relationship between per capita income and chronic conditions, with the poor suffering a greater burden of chronic conditions. Our results indicated a preference for private providers by patients seeking care for chronic conditions among the urban poor in KG Halli. This preference increases when income rises. However, those in the extreme levels of poverty and the elderly still rely on government facilities, indicating a profound schism in the Indian health system. In addition, government facilities are preferred for secondary and tertiary care. Our findings provide further evidence of the urgent need to improve care for chronic conditions among the urban poor, with a preferential focus on improving service delivery in government health facilities.

## Abbreviations

KG Halli: Kadugondanahalli; OR: Odds ratio; UHARP: Urban health action research project.

## Competing interests

UB, BST, RD and CMM are involved in implementation of the Urban Health Action Research Project. The authors declare that they have no competing interests.

## Authors’ contributions

ND and RD conceptualised the overall study (house-to-house survey) and designed the questionnaire. UB, RD, TSB, and CMM supervised the data collection and did data validation. UB and TSB, with inputs from PK, prepared the dataset for analysis. UB conceptualised the analytical approach used in this paper, analysed the data and wrote the draft manuscript. ND, PK, BC, RD and TSB reviewed and commented on the manuscript. UB revised the manuscript. All the authors read and approved the final manuscript submitted to the journal.

## Pre-publication history

The pre-publication history for this paper can be accessed here:

http://www.biomedcentral.com/1472-6963/13/306/prepub
